# Thoracic composite hemangioendothelioma with neuroendocrine marker expression

**DOI:** 10.1186/s40792-021-01331-3

**Published:** 2021-11-27

**Authors:** Ei Miyamoto, Kenji Seki, Hiroyuki Katsuragawa, Yuji Yoshimoto, Yuki Ohsumi, Takamasa Fukui, Masashi Gotoh, Tatsuo Nakagawa

**Affiliations:** 1 Division of Thoracic Surgery, Department of Surgery, Tenri Hospital, 200 Mishimacho, Tenri, Nara 6328552 Japan; 2 Division of Orthopedic Surgery, Department of Surgery, Tenri Hospital, Tenri, Nara Japan; 3 Department of Diagnostic Pathology, Pathologist, Tenri Hospital, Tenri, Nara Japan; 4 Division of Plastic Surgery, Department of Surgery, Tenri Hospital, Tenri, Nara Japan

**Keywords:** Neuroendocrine composite hemangioendothelioma, Chest wall resection, Rare vascular neoplasm

## Abstract

**Background:**

Composite hemangioendothelioma is an extraordinarily rare form of vascular neoplasm which develops predominantly in the skins and soft tissues of the adults. Neuroendocrine marker expression in composite hemangioendothelioma is considered as specifically relevant to the more aggressive behavior.

**Case presentation:**

The patient was a 71-year-old man complaining continuous back pain. Computed tomography (CT) showed that 10 cm of contrast-enhanced soft tissue mass was occurring on the right posterior chest wall and developing adjacent to the spinal canal. Via the laminectomy, the tumor end was identified and separated from the dura mater. Then, via the posterolateral thoracotomy, the en bloc resection was achieved by separating the tumor from the diaphragm and vertebras. Histologic examination showed a complex combination of epithelioid and retiform hemangioendothelioma areas which were positive for anti-synaptophysin staining. At 12-month follow-up, there were no signs of tumor recurrence on CT, and the patient had no symptom.

**Conclusions:**

We achieved the complete resection of a huge thoracic neuroendocrine composite hemangioendothelioma developing adjacent to the spinal canal. The combination of the posterior lumbar laminectomy and the following posterior thoracotomy is a viable approach to radically resect a thoracic neuroendocrine composite hemangioendothelioma involving chest wall.

**Supplementary Information:**

The online version contains supplementary material available at 10.1186/s40792-021-01331-3.

## Introduction

Composite hemangioendothelioma is an extraordinarily rare type of endothelial tumor developing chiefly in the skin and the superficial soft tissue of the adults, showing the distinctive combination of retiform and epithelioid features [1]. Only four cases of thoracic composite hemangioendothelioma have been reported to date [1–4]. Although the prognosis of the patients with composite hemangioendotheliomas has been reported as favorable with a high rate of local recurrence but a low risk of lymph node or distant metastases [1, 5–7], the presence of neuroendocrine marker expression in composite hemangioendothelioma is considered as relevant to the aggressive potentiality [1]. Here, we detail how we achieved the complete resection of a huge thoracic neuroendocrine composite hemangioendothelioma developing deeply inside the right posterior chest wall.

## Case report

**Fig. 1:**
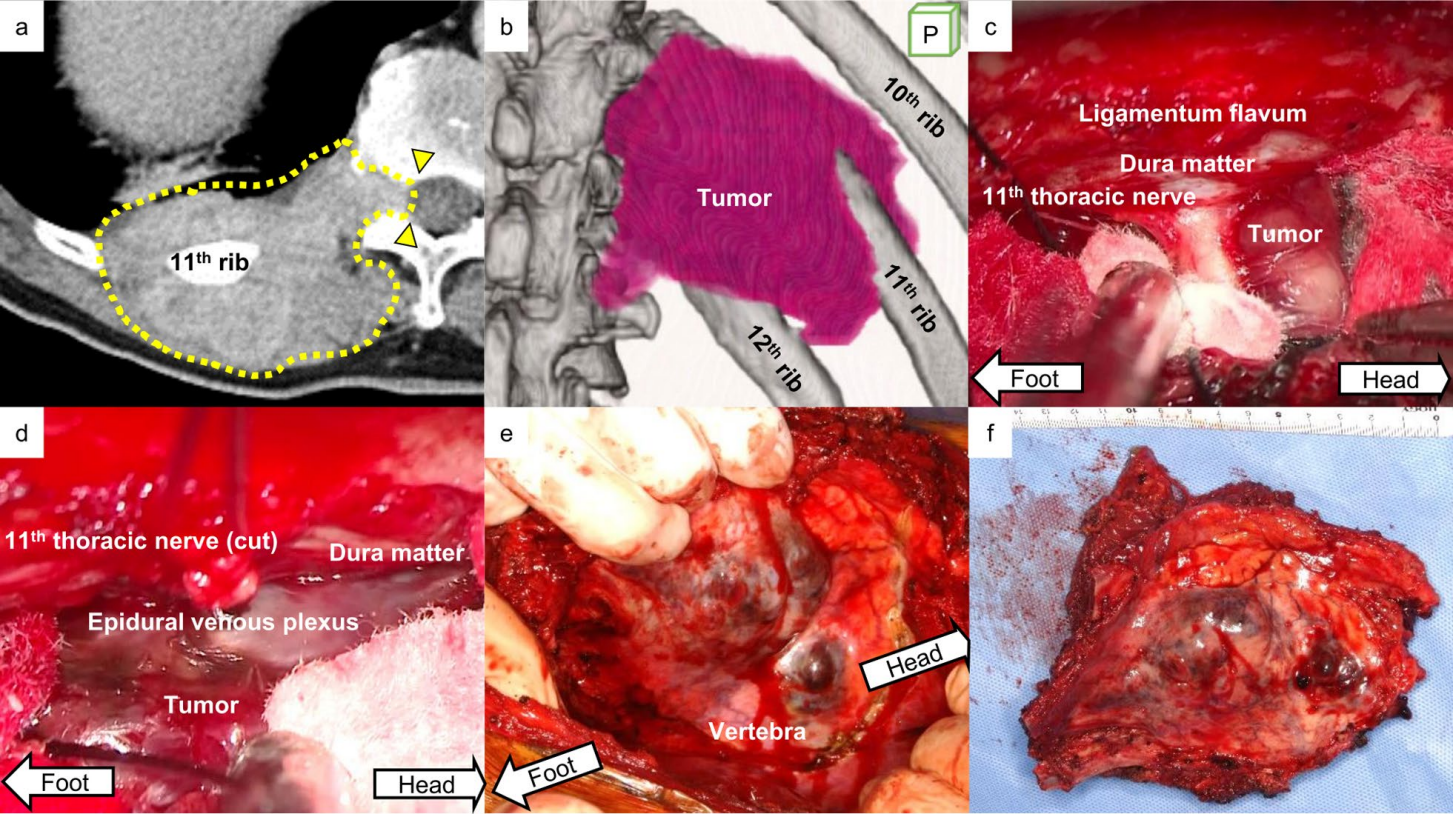
Images of the tumor and vital surgical procedures. **a** An axial view of chest CT. A huge dumbbell shaped tumor (yellow dashed line) was developing into the right Th11–Th12 intervertebral foramen and approaching the spinal canal (yellow arrowheads). **b** A posterior view of the tumor on 3D-CT. The right 11th and 12th ribs were involved in the tumor. **c** Via the posterior microscopic approach, the end of the tumor was identified as reaching the proximity of the dura mater. **d** The tumor was successfully separated from the dura matter. **e** Via thoracotomy, the resection line on the vertebra was carefully determined with surgical margin from the tumor end. **f** En bloc tumor resection

The patient was a 71-year-old man complaining continuous pain on the right back. Imaging studies showed that 10 cm of contrast-enhanced soft tissue mass on the right posterior chest wall was involving the 11th and 12th ribs, and developing to the spinal canal via the Th11–Th12 intervertebral foramen (Fig. 1a, b). The incisional biopsy was suggestive of peripheral nerve tumor or unusual vascular neoplasm with neuroendocrine marker expression.

**Fig. 2:**
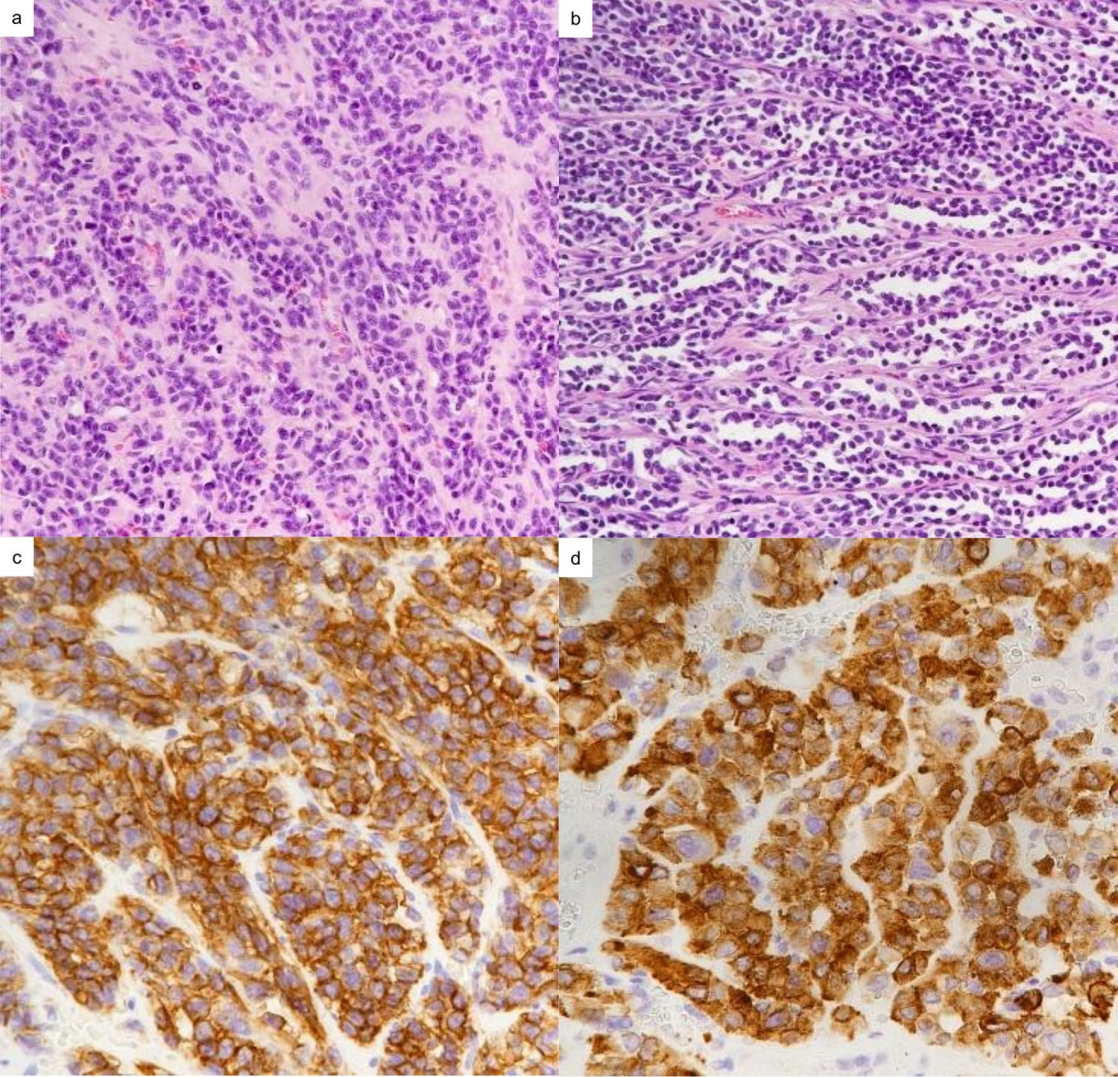
Microscopic findings of neuroendocrine composite hemangioendothelioma. **a** The epithelioid portion of the tumor was consisted of a sheet-like proliferation of uniform, small, epithelioid endothelial cells (H&E, ×20). **b** In the retiform area, the tumor was composed of elongated, branching vascular channels lined by monotonous, hobnailing endothelial cells (H&E, ×20). Thus, this case was diagnosed as composite hemangioendothelioma. The tumor cells showed diffuse and strong immunoreactivity for CD31 (**c**; ×40), and synaptophysin (**d**; ×40). *H&E* hematoxylin and eosin staining

The patient was anesthetized on prone position. Skin incision was determined to perform the posterior spinal fusion and prepare the skin and muscle flap for covering the chest wall defect after the tumor resection, i.e., longitudinal midline skin incision over the spine to expose the right side of the thoracic spine and the second incision along the rib and over the tumor. After splitting the spinalis thoracis muscle, the Th11–Th12 transverse processes and the Th11–L1 spinous processes were cut off at the levels of the base and the lamina was partially removed to approach the Th11 spinal canal. The Th11 thoracic nerve was dissected at the level of the root so that the proximal end of the tumor was clearly identified as developing to the exact proximity of the dura mater (Fig. 1c, d and Additional file 1: Video S1). The tumor end was separated together with the epidural venous plexus from the dura mater so that the resection margin was considered to be free from the tumor, but minimal. Then, the proximal ends of 11th and 12th ribs were separated from the vertebra. The posterior spinal fusion was established to mend Th9–L1 vertebras. Via thoracotomy, the distal portions of the 10th–12th ribs were cut with at least 2 cm of surgical margin and the resection line on the diaphragm and the paravertebral area was determined (Fig. 1e). There were no gross pleural dissemination and direct invasion to the right lung. The tumor mass was carefully separated from the vertebras so that the en bloc resection was achieved (Fig. 1f). The thorax wall defect (Additional file 2: Video S2) was covered with a thick GORE-TEX^®^ sheet and the muscle flap coverings which the latissimus dorsi muscle and the longissimus dorsi muscle had been partially spared for at the beginning of the surgery. Microscopically, the tumor was characterized as an admixture of epithelioid and retiform areas (Fig. 2a, b), suggestive of composite hemangioendothelioma. Tumor cells were focally positive for anti-CD31 (an endothelial marker) and anti-synaptophysin (a marker protein for neuroendocrine cells and neoplasms) immunohistochemistry staining (Fig. 2c, d), but negative for CD56 and chromogranin. The patient was able to walk by himself on postoperative day 1, and the post-surgery course was uneventful. A split-course adjuvant radiotherapy in dose of 50 Gy was applied against the right lateral part of the Th9–L1 vertebras. Chest CT at 12 months after the surgery showed no signs of recurrence.

## Discussion

In the 5th edition of the World Health Organization classification of Tumors of Soft Tissue and Bone, composite hemangioendothelioma is defined as a locally aggressive, rarely metastasizing vascular neoplasm, containing an admixture of histologically distinct components [8]. To our knowledge, this is the first report to demonstrate the successful resection of a thoracic composite hemangioendothelioma with neuroendocrine marker expression which developed deep inside the chest wall. Given the clinical relevance to rapidly developing metastases of the tumor, Perry et al. pointed the possibility that composite hemangioendotheliomas with neuroendocrine marker expression (mostly synaptophysin) might be better interpreted as representing a form of malignant progression in retiform and Dabska-type hemangioendotheliomas which are often positive for synaptophysin, or closely related to angiosarcomas [1]. Whether any distant metastases will develop in the follow-up period of the current case remains to be seen.

In terms of surgical strategy, in the current case, we were able to successfully separate the tumor from the dura matter via the laminectomy, and determine the resection line on the anterolateral part of the vertebra, diaphragm, and chest wall via the following posterolateral thoracotomy. The current approach can be also a viable option for achieving the radical resection of not only thoracic composite hemangioendothelioma, but any other types of soft tissue neoplasms which occur deep in the posterior chest wall and develop adjacent to the vertebra and the spinal canal. An adjuvant radiotherapy is also to be considered against the minimal or positive margins, even though the significance in reducing locoregional recurrence of such rare vascular tumors is unclear.

## Conclusions

Via the posterior lumbar laminectomy and the following posterior thoracotomy, we achieved the complete resection of a huge thoracic neuroendocrine composite hemangioendothelioma developing adjacent to the spinal canal.

## Supplementary Information

**Additional file 1: Video S1.** Microscopic procedures.

**Additional file 2: Video S2.** Macroscopic procedures.

## Data Availability

Not applicable.

## Abbreviations

CT: Computed tomography
H&E: Hematoxylin and eosin staining
